# The Role of HMGB1 Signaling Pathway in the Development and Progression of Hepatocellular Carcinoma: A Review

**DOI:** 10.3390/ijms160922527

**Published:** 2015-09-17

**Authors:** Xuanbin Wang, Longchao Xiang, Hongliang Li, Ping Chen, Yibin Feng, Jingxuan Zhang, Nian Yang, Fei Li, Ye Wang, Quifang Zhang, Fang Li, Fengjun Cao

**Affiliations:** 1 Laboratory of Chinese Herbal Pharmacology, Renmin Hospital, 30 South Renmin Road, Shiyan 442000, Hubei, China; E-Mails: longchaoxiang@hbmu.edu.cn (L.X.); hongliangli@hbmu.edu.cn (H.L.); pingchen@hbmu.edu.cn (P.C.); zhangjx@hbmu.edu.cn (J.Z.); nianyang@hbmu.edu.cn (N.Y.); feili@hbmu.edu.cn (Fei L.); yewang@hbmu.edu.cn (Y.W.); zhangqiufang@hbmu.edu.cn (Q.Z.); fangli@hbmu.edu.cn (Fang L.); fengjuncao@hbmu.edu.cn (F.C.); 2 Hubei Key Laboratory of Wudang Local Chinese Medicine Research, Hubei University of Medicine, 30 South Renmin Road, Shiyan 442000, Hubei, China; 3 School of Chinese Medicine, the University of Hong Kong, 10 Sassoon Road, Pokfulam, Hong Kong, China; E-Mail: yfeng@hku.hk

**Keywords:** high mobility group protein B1, receptor for advanced glycation end product, toll-like receptor, triggering receptor expressed on myeloid cells-1, hepatocellular carcinoma

## Abstract

The story of high mobility group protein B1 (HMGB1) in cancer is complicated and the function of HMGB1 in different cancers is uncertain. This review aims to retrieve literature regarding HMGB1 from English electronic resources, analyze and summarize the role of the HMGB1 signaling pathway in hepatocellular carcinoma (HCC), and provide useful information for carcinogenesis and progression of HCC. Results showed that HMGB1 could induce cell proliferation, differentiation, cell death, angiogenesis, metastasis, inflammation, and enhance immunofunction in *in vitro* and *in vivo* HCC models. HMGB1 and its downstream receptors RAGE, TLRs and TREM-1 may be potential anticancer targets. In conclusion, HMGB1 plays an important role in oncogenesis and represents a novel therapeutic target, which deserves further study.

## 1. Introduction

According to the database from GLOBOCAN 2012, liver cancer has the fifth highest incidence rate and is the second most life-threatening cancer in the world. There were an estimated 14.1 million new cases and 8.2 million cancer deaths worldwide in 2012, among which there were 782,500 new patients and 745,500 deaths caused by liver cancer [1]. Of note, liver cancer ranked the second leading cause of cancer death in males and the sixth in females. Furthermore, hepatocellular carcinoma (HCC) is the primary liver cancer at a dominant rate which is due to the infections of hepatitis B (HBV) and hepatitis C (HCV), food contamination of aflatoxin, alcohol- or nonalcohol-related obesity, type 2 diabetes, cirrhosis, and smoking [1].

In this scenario, it is essential for scientists to pay great affords for HCC therapies. Targeting the hallmarks of cancer is usually one of the approaches to anchoring this issue. For HCC, hallmarks include sustaining proliferative signaling, evading growth suppressors, avoiding immune destruction, enabling replicative immortality, promoting inflammation, activating invasion and metastasis, inducing angiogenesis, mediating genome instability and mutation, resisting cell death and deregulating cellular energetic [2,3]. This means the more hallmarks, the more signaling pathways and cytokines are involved. Interestingly, however, high mobility group box 1 (HMGB1), a protein involving in many signaling pathways, has discovered and been investigated since 1973 [4].

HMGB1 is one of the HMGB family members (HMGB 1, 2, 3 and 4) with a molecular weight of 25 to 30 kDa [5], which consists 215 amino acids and three domains. The three domains include HMGB A box (9–79 aa), HMG B box (95–163 aa) and the C-terminal acidic tail (186–215 aa) [4]. In normal organs, HMGB1 acts as a positive factor to protect cells from injury. For instance, the model mice were most likely to be sensitive to liver ischemia/reperfusion [6], pancreatitis [7] and sepsis [8] if HMGB1 was knocked out in the liver, pancreas or macrophages, respectively. In contrast, HMGB1 functions as one of the damage-associated molecular patterns (DAMPs) in the sterile inflammation model by amplifying hepatic ischemia/reperfusion and acetaminophen-induced liver necrotic injury [9]. Moreover, HMGB1 has been demonstrated as a critical role in a number of cancers, including colorectal [10], breast [11,12], lung [13,14,15,16], prostate [17], cervical [18], skin [19], kidney [20,21], gastric [22,23,24,25,26], pancreatic [27,28,29], osteosarcoma [30] and leukemia [31]. As to the signal pathways of HMGB1, its receptors include receptor for advanced glycation end product (RAGE) [32,33], the toll-like receptors (TLRs, such as TLR-2, 4 and 9) [34,35,36,37], intergrin [38], α-synuclein filaments [39], proteoglycans (e.g., heparin sulfate [40]), CD24 [41], the T-cell immunoglobulin domain and mucin domain-3 (TIM-3) [42], the member of the G protein-coupled receptors CXCR4 [43], *N*-methyl-d-aspartate receptor (NMDAR) [44] and the triggering receptor expressed on myeloid cells-1 (TREM1) [45].

HMGB1 is expressed in all eukaryotic cells and highly conserved through evolution. There is 99% identity of the *hmgb1* gene in mammals [46]. To date, few research papers have reported its mutant, whereas the modifications and regulations are critical for HMGB1 location and function: Acetylated HMGB1 in cancer cells participates DNA replication; ADP-ribosylation of HMGB1 regulates cell death; methylation of HMGB1 facilitates its translocation from nucleus to the cytoplasm; phosphorylation of HMGB1 affects both its DNA-binding activity and nucleo-cytoplasmic distribution and release. In addition, location and function of HMGB1 highly depend on its redox status. Disulfide HMGB1 exhibits cytokine-inducing activity while sulfonyl HMGB1 has nonimmune activity [4].

However, the functions of HMGB1 in cancer are complicated and paradoxical because of its difference of intracellular and extracellular locations. In recent research, it has been documented as a regulator for a number of DNA events, cell differentiation, inflammatory response, cell migration, cell proliferation, cell deaths, cellular senescence, microRNA biogenesis, immune response, tissue regeneration, antibacterial, and so on [4], whereas its actions and the underlying mechanisms on the oncogenesis and advance in HCC are still unclear.

Here, we reviewed the effects of HMGB1 on the oncogenesis and progression in HCC. The review aims to critically summarize the multiple functions of HMGB1, the receptors and the signaling pathways to unveil the hallmarks and the potential therapeutic targets of HCC.

## 2. HMGB1 and Cell Proliferation in HCC 

For HCC, some cell cycle proteins and proliferation cytokines are central in the proliferation, such as cyclin D1 and proliferating cell nuclear antigen (PCNA). HMGB1 not only increased cyclin D1 and PCNA to induce the proliferation of HCC [47], but also regulates for tumor multiplicity and size, alpha-fetoprotein (AFP) level and advanced TNM stage in HCC [48], indicating HMGB1 could be used as a biomarker for HCC diagnosis which deserves further study [49].

The signaling pathways for HMGB1 on the proliferation in HCC may involve its downstream, RAGE and TLRs. On the one hand, HMGB1 could increase the cellular proliferation by HMGB1/RAGE/NF-κB pathway. Knockdown of RAGE by siRNA inhibited cell growth in HCC *in vitro* [50,51]. Furthermore, the serum level of RAGE in the primary hepatocellular carcinoma (PHC) tissue was higher than that of the adjacent para-neoplastic liver samples [51], indicating that a critical role and a novel potential target of RAGE in HCC [50,52]. On the other hand, under the condition of hypoxia caused by the rapid growth of HCC, HMGB1 was translocated from the nucleus to the cytosol and bound mitochondrial DNA (mtDNA) in the cytoplasm of hypoxic tumor cells, inducing tumor growth by activating TLR-9 signaling pathways both *in vitro* and *in vivo* [53].

## 3. HMGB1 and Angiogenesis in HCC 

Recently, HMGB1 has been recognized as a pro-angiogenesis factor leading to the generation of vascular endothelial growth factor (VEGF) in colon cancer [54,55], while RAGE was identified as the requirement for cell angiogenesis in HCC [56]. Since RAGE is known as one of the receptors of HMGB1, this indirectly indicates that HMGB1 may induce angiogenesis by RAGE in HCC. Unfortunately, however, to date, the direct evidence of HMGB1 in the angiogenesis of HCC has not been reported.

## 4. HMGB1 and Cell Death in HCC

Autophagy and apoptosis are recognized as both the programmed cell deaths. In HCC, the release of HMGB1 from nuclei to cytoplasm was reported as an inducer for cell autophagic cell death, which may be associated with ROS and/or Beclin-1. In contrast, when this pathway was inhibited by administration of antioxidant *N*-acetyl-cysteine (NAC), the cell death was altered from autophagic to apoptotic [57]. Such a role of HMGB1 might be partially proved by another research area: Ethyl pyruvate (VP) induced apoptosis by decreasing HMGB1 [32]. However, interestingly, autophagic cell death could exist in an HMGB1-independent way since there were no significantly changes of baseline and glucocorticoid-induced hepatic gene expression with HMGB1 ablation [58,59]. HMGB1 also induced HCC cell apoptosis by inhibition of p38-dependent mitochondrial pathway [60], as HMGB1 could be released from necrotic cells [61]. This may result from the complicate multiple functions of HMGB1 partially in a cross-talk way [4,61] or the different targeting strategies between the different studies [62].

## 5. HMGB1 and Cell Differentiation in HCC

As a nuclear non-histone protein response to various stimuli, HMGB1 is elucidated to be involved in the differentiation of HCC. HMGB1 was a higher level in HCC tissues than that in the normal tissues. For moderately differentiated cancer cells, the localization of HMGB1 was perinuclear. In contrast, in the low differentiated cancer cells, HMGB1 normally resided in the nucleus [63]. In addition, the role of HMGB1 in HCC differentiation was associated with its downstream receptor. Indeed, similar to HMGB1, the high level of RAGE was required for hepatitis as well as HCC. Furthermore, RAGE exhibited a higher level in well- and moderately differentiated HCC but declined as tumors dedifferentiated to poorly differentiated HCC [64]. As HCC resistance to hypoxia was found to have higher levels of RAGE, RAGE transfectant significantly prolonged cell availability under hypoxia, indicating the early stage of oncogenesis with less oxygen and nutrition may acquire resistance by HMGB1/RAGE axis [64].

## 6. HMGB1 and Metastasis in HCC

To date, strategies for HCC radiotherapie, chemotherapie and surgical resection are inefficient due to high recurrence rate and metastasis. However, the recurrence after tumor rectomy at least partially stems from the distant metastasis to other tissues and organs [65]. HMGB1 was attributed to the one of the causes for HCC metastasis [49], and down-regulation of HMGB1 by siRNA led to the decrease of migration and invasion [66]. HMGB1 repressed the matrix metalloproteinase (MMP) inhibitors RECK and TIMP3, which induced the expression of MMPs to enable metastases [67]. The main signaling pathways at least include: (1) The activation of HMGB1 required the up-stream signaling pathway of HSP70/Beclin-1 [68]. In contrast, HMGB1 could be inhibited by PPARγ agonists [50]; (2) HMGB1 activated RAGE/NF-кB [63,69] and/or TLR-4/caspase-1 [36] for HCC metastasis; (3) HMGB1 also mediated the activation of metastasis by inducing IL6/Stat3-miR-21 axis [67]. Of note, HMGB1-mediated metastasis of HCC is one of the inflammatory responses to hypoxia stress [36].

## 7. HMGB1 and Inflammatory Response in HCC

HCC is definitely associated with hepatitis B and hepatitis C and other sterile liver injury such as alcoholic fatty liver disease (AFLD) [1]. With regard to inflammation response, HMGB1 functions as an inducer to activate macrophages and leukocytes, as well as a number of inflammatory cytokines [61]. In HBV transgenic mice, HMBG1 may be involved in the amplification of the cytotoxic T lymphocytes (CTLs)-initiated liver damage [70], while in sterile inflammation, HMGB1 can be released from necrotic cells and triggered neutrophil-mediated liver injury [9], suggesting that treatment of HMGB1 inhibitors may be a potential strategy for fighting against hepatitis-caused HCC. In HCC, p53 promotes inflammation-associated hepatocarcinogenesis by inducing HMGB1 release although p53 is usually thought as a tumor suppression factor [71]. The constant activation of p53 could induce pro-tumorigenic inflammation, at least in part, via inducing HMGB1 release. By contrast, application of HMGB1 inhibitors when restoring p53 in cancer therapy might protect against pro-tumorigenic effects while leaving p53-mediated clearance of cancer cells intact [71], indicating the active function of the p53-binding domain in HMGB1 protein. In addition, RAGE, NF-кB and TREM-1 play a great role in HMGB1-induced inflammation [45,72,73]. Therefore, HMGB1 is an important target for the alternation from hepatitis to tumor or early hepatocarcinogenesis.

## 8. HMGB1 and Immune Function in HCC

The immune system including the innate and adaptive parts protects the body against pathogens, destroy cancer cells and foreign substances. In some case, HMGB1 regulates innate and adaptive immune responses by TLRs and RAGE pathways [4,74], guiding repair and immune protection at the site of tissue injury. In injury tissue, HMGB1 is passively released by necrotic cells and actively secreted by monocytes or macrophages cells [5,46]. HMGB1 is also secreted in the CTLs-killed cells [75]. In addition, HMGB1 attracts inflammatory leukocytes into paracetaminophen-mediated massive necrotic liver [76]. Of note, HMGB1 may interact with its receptor, TLR-9 and enhance the TLR-9-dependent immunostimulatory effect of CpG DNAs on macrophages and dendritic cells (DCs) [5]. These implicate the crucial role of HMGB1 in innate immunity. On the other side, HMGB1 mediates T-cell-dependent acquired immune response acting as an adaptive immune adjuvant [5]. HMGB1-containing proteins may also be used as a tumor-derived autophagosome vaccine for antitumor [77].

However, the role of HMGB1 in liver cancer immunity needs to be further studied. For the patients who suffered from HCC and had undergone transarterial chemoembolization (TACE) therapy, though the level of HMGB1 was found to increase after TACE, the level of HMGB1 was no different between the “progression group” and “no progression group” patients after TACE for 24 h. In contrast, the soluble receptor of advanced glycation end products (sRAGE) were significantly higher in the non-progression group than that in the progression group [33], indicating the clinical prognosis value of sRAGE was superior to HMGB1 for patients undergoing TACE therapy. 

## 9. Discussion

HMGB1 has attracted researchers since it was discovered in 1973, because it plays a critical role in various diseases and disorders, especially in inflammatory, immune responses, and hypoxia in cancer microenvironment. However, little is known about its role in HCC.

**Figure 1:**
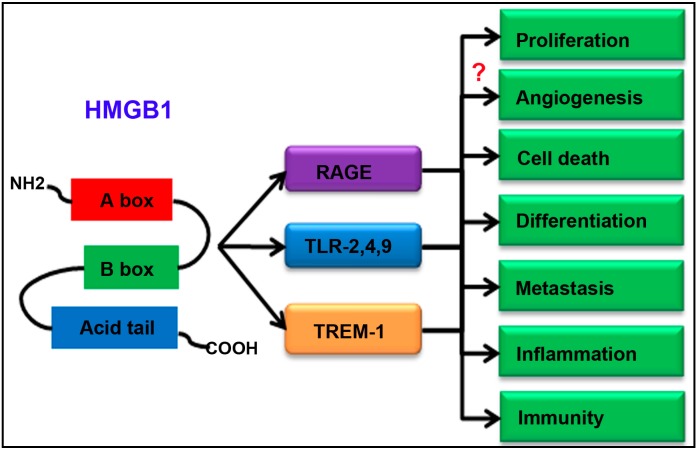
Schematic diagram of high mobility group protein B1 (HMGB1) signaling pathways in hepatocellular carcinoma (HCC). “?” represents that the more evidence is required.

In this review, we summarized HMGB1 in oncogenesis and progression in HCC. Indeed, HMGB1 can induce cell proliferation, angiogenesis and metastasis, cell death, differentiation and inflammation (Table 1). While HMGB1 enhancing immunofunction after TACE may be not prognostic value for TACE [33]. As to the underlying signaling pathways, HMGB1 receptors in HCC include RAGE, TLRs and TREM-1 [45,72,73], though there are a number of receptors of HMGB1 in other cancers. The schematic diagram of HMGB1 signaling pathways in HCC is illustrated in Figure 1.

However, there are conflicting roles of HMGB1 acting as both a tumor suppressor and an oncogenic factor in cancer. On the one hand, in the transcriptional level, p53, the tumor suppressor, could down-regulate the activity of the HMGB1 gene promoter by binding to CTF2. In contrast, CTF2 was proved both to up-regulate the promoter of p21 by binding to p53 and to down-regulate the promoter of p21 by binding to p73, a homologue of p53 [78], which indicated an opposite transcriptional regulation of HMGB1. On the other hand, for the role in oncogenesis and progression of tumor, HMGB1 also had paradox effects. For example, as to the pro-tumor roles, HMGB1 induced the tumorigenesis such as sustenance of cell proliferation, differentiation, angiogenesis, metastasis, inflammation, and enhanced immunofunction in *in vitro* and *in vivo* HCC models. However, according to the research, intercellular HMGB1 also could interact with RB, a tumor suppressor in breast cancer [79], and increase genome instability and autophagy [80]. This may depend on the context and the study conditions as well as HMGB1 location and modification [32,60,81]. However, whether the roles of HMGB1 in the carcinogenesis and progression in HCC are good or bad, is still to be studied comprehensively in the future.

Additionally, more details of HMGB1 about its up- and down-regulation, alteration between different signaling pathways as well as strategies must constitute future potential approaches.

**Table 1:** HMGB1 and its roles in HCC.

Location of HMGB1	Function	Receptor	References
recombinant	induce proliferation	NA	[47]
NA	promote progression	NA	[48]
NA	promote metastasis	NA	[49]
NA	induce proliferation and metastasis, block apoptosis	RAGE	[50]
NA	induce proliferation	RAGE	[51]
NA	induce proliferation and invasion	RAGE	[52]
cytosol	induce proliferation	TLR-9	[53]
NA	induce angiogenesis	RAGE	[56]
cytosol	induce autophagic cell death	NA	[57]
NA	induce proliferation and reduce apoptosis	RAGE	[32]
NA	induce apoptosis	NA	[60]
perinuclear	induce differentiation	NA	[63]
nucleus	block differentiation	NA	[63]
NA	induce differentiation	RAGE	[64]
NA	induce metastasis	NA	[66]
NA	induce metastasis	NA	[67]
NA	induce metastasis	RAGE	[68]
NA	induce metastasis	RAGE	[69]
NA	induce metastasis	TLR-4 and RAGE	[36]
NA	induce metastasis	NA	[67]
NA	induce inflammation	NA	[71]
NA	induce inflammation	TREM-1	[45]
NA	induce inflammation	RAGE	[72]
NA	induce inflammation	TREM-1 and RAGE	[73]
NA	induce immunity	RAGE	[33]
NA	induce proliferation and metastasis	NA	[82]
serum	induce carcinogenesis	NA	[83]
NA	induce carcinogenesis	NA	[84]
NA	induce proliferation and metastasis	NA	[85]
serum	induce metastasis	TLR-4	[86]
recombinant	induce proliferation and metastasis, reduce apoptosis	TLR-2	[87]

## 10. Conclusions

In summary, HMGB1 plays a pivotal role in oncogenesis and progression in HCC which may be a potential target for therapies and is worthy of further study. 
